# A genome-wide systems analysis reveals strong link between colorectal cancer and trimethylamine N-oxide (TMAO), a gut microbial metabolite of dietary meat and fat

**DOI:** 10.1186/1471-2164-16-S7-S4

**Published:** 2015-06-11

**Authors:** Rong Xu, QuanQiu Wang, Li Li

**Affiliations:** 1 Center for Clinical Investigation, Medical Informatics Program, Case Western Reserve University, 2103 Cornell Road, 44106 Cleveland, USA; 2 ThinTek LLC, 94306 Palo Alto, USA; 3 Departments of Family Medicine and Community Health, Epidemiology and Biostatistics, Case Western Reserve University, Cleveland, USA

**Keywords:** systems biology, network medicine, colorectal cancer, trimethylamine N-oxide (TMAO), human gut microbiome, dietary meat and fat

## Abstract

**Background:**

Dietary intakes of red meat and fat are established risk factors for both colorectal cancer (CRC) and cardiovascular disease (CVDs). Recent studies have shown a mechanistic link between TMAO, an intestinal microbial metabolite of red meat and fat, and risk of CVDs. Data linking TMAO directly to CRC is, however, lacking. Here, we present an unbiased data-driven network-based systems approach to uncover a potential genetic relationship between TMAO and CRC.

**Materials and methods:**

We constructed two different epigenetic interaction networks (EINs) using chemical-gene, disease-gene and protein-protein interaction data from multiple large-scale data resources. We developed a network-based ranking algorithm to ascertain TMAO-related diseases from EINs. We systematically analyzed disease categories among TMAO-related diseases at different ranking cutoffs. We then determined which genetic pathways were associated with both TMAO and CRC.

**Results:**

We show that CVDs and their major risk factors were ranked highly among TMAO-related diseases, confirming the newly discovered mechanistic link between CVDs and TMAO, and thus validating our algorithms. CRC was ranked highly among TMAO-related disease retrieved from both EINs (top 0.02%, #1 out of 4,372 diseases retrieved based on Mendelian genetics and top 10.9% among 882 diseases based on genome-wide association genetics), providing strong supporting evidence for our hypothesis that TMAO is genetically related to CRC. We have also identified putative genetic pathways that may link TMAO to CRC, which warrants further investigation. Through systematic disease enrichment analysis, we also demonstrated that TMAO is related to metabolic syndromes and cancers in general.

**Conclusions:**

Our genome-wide analysis demonstrates that systems approaches to studying the epigenetic interactions among diet, microbiome metabolisms, and disease genetics hold promise for understanding disease pathogenesis. Our results show that TMAO is genetically associated with CRC. This study suggests that TMAO may be an important intermediate marker linking dietary meat and fat and gut microbiota metabolism to risk of CRC, underscoring opportunities for the development of new gut microbiome-dependent diagnostic tests and therapeutics for CRC.

## Introduction

Colorectal cancer (CRC) represents the second most common cause of cancer in women (9.2%) and the third most common in men (10.0%). Diet clearly plays an important role in colon carcinogenesis. The Western diet, characterized by high fat and meat consumption, has been associated with increased risk of colorectal cancer in a large number of epidemiological studies [1-3]. The risk association is particularly strong for red meat intake. In effect, an extensive review of the existing evidence by an international panel of experts concluded that a high intake of red meat is a convincing and probable cause of colorectal cancer [4].

The complex gut microbiota harbored by individuals have long been proposed to play an important role in colon carcinogenesis [5-7]. Recent studies comparing patients with colorectal neoplasia and healthy controls have found differences either in the relative abundance of certain microbial species or in the taxonomic composition of the microbiome. In particular, three studies using high throughput sequencing to characterize the composition of microbiota have discovered enrichment of Fusobacterium species in human colorectal tumors or adenomas as compared to matched normal control tissues, providing direct evidence for a link of gut microbiome to colorectal cancer [8-10]. The exact mechanisms by which gut microflora may modulate colorectal cancer risk, however, remain largely unexplored.

Recent studies have discovered that trimethylamine N-oxide (TMAO) generated by gut microbiota metabolism of dietary L-carnitine, a trimethylamine abundant in red meat, and dietary phosphatidylcholine is mechanistically linked to risk of cardiovascular diseases (CVDs) [11-14]. It is further shown that human gut microbiota are required to form TMAO from dietary red meat and fat, and specific bacterial taxa are associated with both plasma levels of TMAO and dietary meat and fat intakes. These studies suggest a novel mechanism involving a complex interplay of human gut microbial community and diet for the observed relationship between dietary red meat and fat consumption and cardiovascular disease.

Whether TMAO plays a similar role in colon carcinogenesis has not been explored. Given the striking similarity of colorectal cancer and cardiovascular diseases in risk association with dietary red meat/fat intakes, we hypothesize that TMAO is an intermediate marker linking dietary red meat and fat and gut microbial metabolism to colorectal cancer. Here, we represent a genome-wide systems approach to the discovery of the genetic links between CRC and TMAO by reasoning over vast amounts of disease-gene association, protein-protein interaction and chemical-gene association data from multiple databases using advanced network-based ranking algorithms.

## Materials and methods

The experimental framework consists of the following steps: (1) we constructed two different genetic disease networks (GDNs) using disease-gene and protein-protein interaction data from multiple large-scale data resources; (2) we modeled the epigenetic interactions between TMAO and diseases by transforming GDNs into epigenetic interaction networks (EINs); (3) we developed a network-based ranking algorithm to find TMAO-related diseases from GDNs. These diseases share a high degree of genetic similarities with TMAO; (4) we validated recent findings that TMAO is associated with cardiovascular diseases; (5) we tested our hypothesis that TMAO might be genetically linked to CRC; (6) we systematically analyzed disease categories among TMAO-related diseases at different ranking cutoffs; and (7) we determined which genetic pathways were associated with both TMAO and CRC.

### Construct genetic disease networks (GDNs)

#### Construct GDN based on OMIM genetics (GDN_OMIM)

We constructed two separate GDNs using disease-gene association data from two complementary data resources. The first one is the Online Mendelian Inheritance in Man (OMIM), a comprehensive database of human genes and genetic phenotypes mainly for rare Mendelian genetic disorders [15]. We downloaded the OMIM database and mapped gene names to their corresponding approved human gene symbols as defined by the HUGO Gene Nomenclature Committee (HGNC) [16]. We extracted a total of 15,462 disease-gene pairs from the OMIM database, representing 5,983 diseases and 8,831 genes. On GDN_OMIM, two diseases were connected if their associated genes (proteins) interact. The edge weights were determined by the numbers of protein-protein interaction (PPI) pairs between two diseases (*D*_*i *_and *D_j _*) and is defined as: $W_{D_{i}D_{j}}=\overset{n}{\underset{k=\text{1}}{\sum }}G_{i}k\overset{m}{\underset{l=\text{1}}{\sum }}G_{jl}$, where *G_i_k *is a gene associated with *D_i_, G_j _l *is a gene associated with *D_j _*, and *G_i_k *is the same as or interacts with *G_j _l *according to known protein-protein (PPI) association data. The PPI data was obtained from the STRING database, a database of known and predicted protein interactions [17]. Currently, STRING contains 5,214,234 proteins from 1,133 organisms. From the STRING database, we obtained a total of 4,137,054 human PPI pairs representing 17,756 human proteins.

#### Construct GDN based on GWAS genetics (GDN_GWAS)

The second source of disease genetics we utilized in constructing GDNs was the Catalog of Published Genome-Wide Association Studies from the US National Human Genome Research Institute (NHGRI), an exhaustive source containing the description of diseaseand trait-associated single nucleotide polymorphisms (SNPs) from published GWAS data [18]. Different from diseases in the OMIM database, diseases in the GWAS catalog are mainly common complex diseases. We first mapped SNPs to their associated strongest genes, which were subsequently mapped to their corresponding approved human gene symbols as defined by the HGNC. In total, we obtained 22,470 disease/trait-gene pairs, representing 881 diseases/traits and 8,689 genes. On GDN_GWAS, two diseases were connected if their associated genes (proteins) interact and the edge weights were the numbers of PPI pairs between two diseases as described above. In summary, the disease network GDN_OMIM consisted of 4,848 nodes and 882,751 edges; GDN_GWAS consisted of 882 nodes and 200,758 edges. Compared to GDN_GWAS, GDN_OMIM contained significantly more diseases, but fewer edges between any two nodes.

### Model the epigenetic interactions between TMAO and diseases by transforming disease networks into epigenetic interaction networks (EINs)

We modeled the epigenetic interactions between TMAO and diseases on both GDN_OMIM and GDN_GWAS by inserting TMAO into these two disease networks. We obtained human genes associated with TMAO from STITCH, a publicly available database of known and predicted interactions of chemicals and proteins [19]. Currently, STITCH contains interactions for between 300,000 small molecules and 2.6 million proteins from 1,133 organisms, with each interaction associated with a score measuring the evidence of the association. In STITCH, TMAO is associated with a total of 553 genes from 932 species, including 54 genes from humans. Table 1 shows ten human genes associated with TMAO.

**Table 1:** Ten TMAO-associated human genes.

Gene Symbol	Gene name
MBD2	Methyl-CpG binding domain protein 2
FMO3	Flavin containing monooxygenase 3
RORC	RAR-related orphan receptor C
SGCG	Sarcoglycan, gamma (35kDa dystrophin-associated glycoprotein)
PNKD	Paroxysmal nonkinesigenic dyskinesia
RNASE1	Ribonuclease, RNase A family, 1 (pancreatic)
NKRF	NFKB repressing factor
PFKM	Phosphofructokinase, muscle
MOCS1	Molybdenum cofactor synthesis 1
TRA2A	Transformer 2 alpha homolog

**Figure 1:**
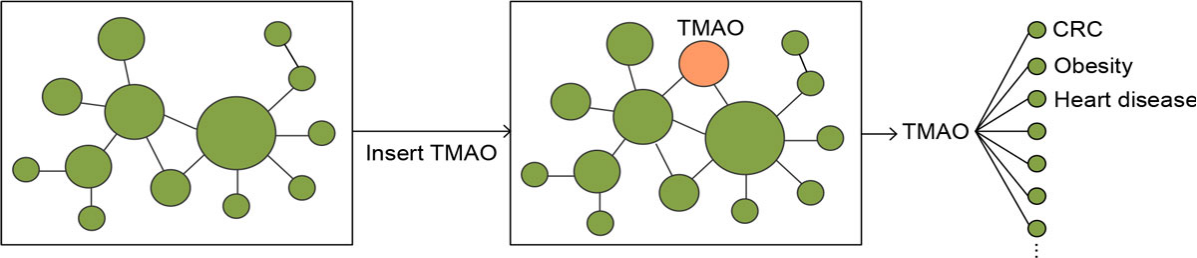
**Finding TMAO-related diseases**. Transforming GDNs into epigenetic interaction network (EINs) and finding TMAO-related diseases from transformed EINs.

We first inserted a pseudo-node representing TMAO into GDNs. This node was then connected to disease nodes on GDNs if TMAO-associated genes interact with disease-associated genes. The edge weights were determined by the numbers of interacting genes between the newly inserted node (*T*) and existing disease nodes (*D_j_*) and is defined as: $W_{Dj}=\overset{n}{\underset{k=1}{\sum }}G_{T}k\overset{m}{\underset{l=1}{\sum }}G_{jl}$ where *G_T _k *is a gene associated with TMAO, *G_j _l *is a gene associated with *D_j _*, and *G_T _k *is the same as or interacts with *G_j _l*. The intuition is that: if TMAO-associated genes participate in the same pathways as disease-associated genes, we can hypothesize that TMAO may be involved in disease pathogenesis. The degree of relatedness between TMAO and diseases was determined by the numbers of interacting gene pairs. After inserting the node "TMAO", we re-normalized the matrices represented by EINs and applied an existing network-based ranking algorithm to find diseases that share high genetic similarities with TMAO (Figure 1). The network-based ranking algorithm was used to find diseases that are related to TMAO both directly and indirectly by taking into account of inter-relationships among diseases. After this step, we created two EINs: the EIN_OMIM was created based on GDN_OMIM and the EIN_GWAS was created based on GDN_GWAS.

### Develop network-based ranking algorithm to find diseases that share high genetic similarities with TMAO

We then developed a network-based ranking algorithm to prioritize diseases on EINs based on their genetic commonalities with TMAO. We retargeted the TopicSensitive PageRank (TSPR) algorithm to rank similar diseases for a given input (TMAO in our study). TSPR is a context-sensitive ranking algorithm for web searches developed by Taher Haveliwala [20]. Versions of this approach have been used in prioritizing disease genes using networks consisting of same node types (i.e. diseases or genes) [21,22]. In this study, we applied the same algorithm to a heterogeneous network consisting of disease nodes and chemical nodes (TMAO in this study) in order to find TMAO-related diseases. The iterative networkbased ranking algorithm in finding similar diseases to a given input is defined as: *p*_*t*+1 _= (1 *− r*)*M p_t _*+ *rp*_0_, wherein *M *is the column-normalized adjacency matrix of EINs, *γ *is a preset probability of restarting from the initial seed node (*γ *= 0.1 in this study), and *p^t ^*is a vector in which the *i_th _*element holds the normalized ranking score of disease *i *at *t_th _*iteration. The initial probability vector *p*^0 ^contains normalized probability for input. In our study, *p*^0 ^contains TMAO, with a probability of 1.0. Diseases are then ranked according to the value in the steady-state probability vector, which is obtained by iterating the algorithm until the change between *p*^*t*+1 ^and *p*^*t *^is less than 10^6^.

### Validate recent findings that TMAO is associated with cardiovascular diseases

Recent studies indicate that high levels of TMAO in the blood are associated with an increased risk of cardiovascular diseases [11-14]. We examined the rankings of cardiovascular diseases and their major risk factors, including high blood cholesterol and triglyceride, high blood pressure, diabetes, and obesity, among diseases retrieved from EINs using TMAO as seed. As positive controls, these diseases are expected to rank highly among TMAO-related diseases.

### Test our hypothesis that TMAO may be genetically associated with CRC

In order to provide evidence supporting our hypothesis that TMAO may be involved in CRC pathogenesis, we tested whether CRC would rank highly among TMAOrelated diseases retrieved from both EINs. High rankings of CRC would imply that TMAO and CRC share high genetics and that TMAO might be associated with CRC carcinogenesis.

### Analyze diseases enriched among top-ranked TMAO-related diseases

To better understand TMAO-related diseases, we determined the kinds of diseases that were enriched among top-ranked diseases retrieved from EINs. We classified diseases into different categories using the 10th revision of the International Statistical Classification of Diseases and Related Health Problems (ICD10), a disease classification scheme designated by the World Health Organization (WHO) [23]. The ICD10 includes 22 highest-level disease classes. We used 16 of the 22 chapters and excluded six non-specific disease classes. Since the terms used in ICD10 may be different from those used in EINs, we mapped disease terms in ICD10 to their synonyms through the unified medical language system (UMLS) unique concept identifiers [24]. Disease chapters and the numbers of diseases in each chapter are listed in Table 2.

**Table 2:** Sixteen disease chapters (classes) and numbers of diseases in each chapter.

Disease Class	Diseases (n)	Disease Classes	Diseases (n)
Certain infectious and parasitic dis- eases	11,598	Diseases of the circulatory system	5544
Neoplasms	14,158	Diseases of the respiratory system	3156
Diseases of the blood and blood forming organs and certain disorders involving the immune mechanism	3264	Diseases of the digestive system	5960
Endocrine, nutritional and metabolic diseases	5438	Diseases of the skin and subcutaneous tissue	4390
Mental and behavioural disorders	6162	Diseases of the musculoskeletal system and connective tissue	11520
Diseases of the nervous system	5258	Diseases of the genitourinary system	5247
Diseases of the eye and adnexa	3735	Congenital malformations, deformations and chromosomal abnormalities	9064
Diseases of the ear and mastoid process	1815	Certain conditions originating in the perinatal period	3454

Since EIN_OMIM contains 4,848 disease nodes and EIN_GWAS contains 882 nodes, we performed disease class enrichment analysis on TMAO-related diseases retrieved from EIN_OIMIM only. For diseases ranked at 10 different ranking cutoffs (top 10%, 20%, . . . 100%), we calculated percentages of the sixteen ICD10 disease classes among them.

### Identify genetic pathways linking TMAO to CRC

**Figure 2:**
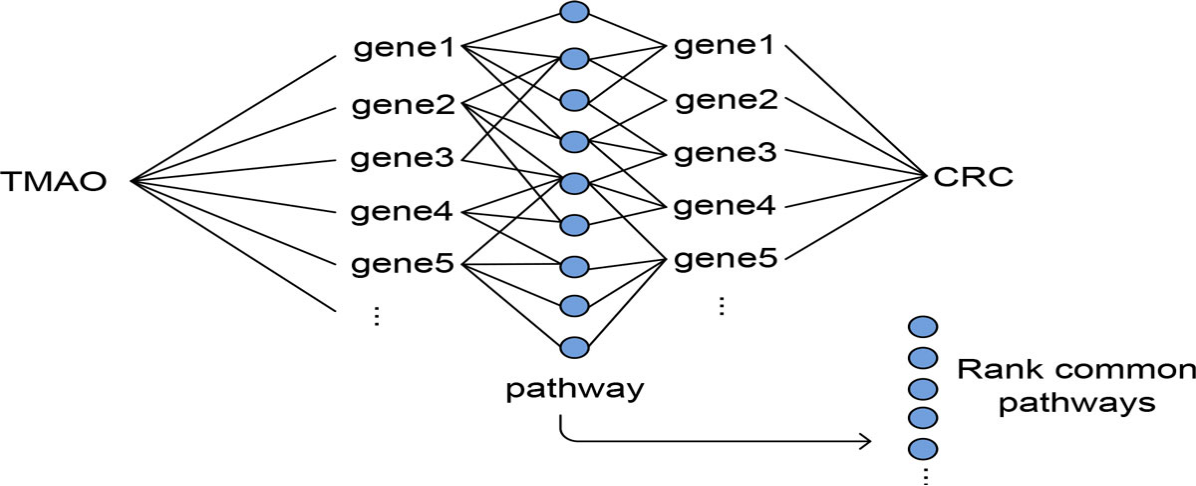
**Finding interplaying genetic ways for TMAO and CRC**. Finding putative genetic pathways linking TMAO to CRC.

In order to gain insights into common mechanistic relationships shared between TMAO and CRC, we identified and ranked genetic pathways linking them (Figure 2). Functions of highly enriched pathways might provide insights into common molecular mechanisms linking TMAO to CRC. TMAO is associated with 54 human genes based on the STITCH database. CRC is associated with 65 genes according to the GWAS catalog and 53 genes according to the OMIM database. There is no direct overlap between CRC-associated genes from OMIM and those from the GWAS catalog. In addition, there is no overlap between TMAO-associated genes and CRC-associated genes. We analyzed gene-associated pathways using the pathway data (a total of 10,295 pathways and gene sets) from the Molecular Signatures Database (MSigDB), a collection of annotated genetic pathways or gene sets from multiple sources [25]. We ranked these pathways based on the numbers of genes associated with TMAO or CRC: $R_{pathway}=\overset{n}{\underset{i=\text{1}}{\sum }}G_{i}$, where *G_i _*is a TMAOor CRC-associated gene that a given pathway contains. We then identified pathways that contain both TMAOand CRC-associated genes and ranked them base on the number of interacting gene-gene pairs between TMAO genes and CRC genes: $R_{common\text{\_}pathway}=\overset{n}{\underset{i=\text{1}}{\sum }}G_{i}\overset{m}{\underset{j=\text{1}}{\sum }}G_{j}$ where *G_i _*is a TMAO-associated gene and *G_j _*is a CRC-associated gene.

## Results

### Cardiovascular diseases (CVDs) are genetically related to TMAO

Recent studies indicate that high levels of TMAO in the blood are associated with an increased risk of CVDs. Our results demonstrated that CVDs as well as their major risk factors, including high blood cholesterol and triglyceride, high blood pressure, diabetes, and obesity, were ranked highly among TMAO-related diseases retrieved from both EIN_OMIM and EIN_GWAS. We retrieved a total of 878 diseases/traits from EIN_GWAS, among which *obesity-related traits *was ranked at top 1 (top 0.11%) and *coronary heart disease *at top 7 (top 0.80%). Other CVDrelated risk factors, including *HDL cholesterol *(top 1.13%), *type 2 diabetes *(top 1.48%), *LDL cholesterol *(top 1.82%), and *metabolic syndrome *(top 3.64%) were also ranked highly (Table 3).

**Table 3:** Top 10 ranked cardiovascular diseases and its related risk factors.

Diseases/traits Based on GWAS genetics (878)		Diseases Based on OMIM genetics (4732)	
Diseases/traits	Ranking (top%)	Diseases	Ranking (top%)
Obesity-related traits	0.11%	Myocardial infarction, susceptibility to	0.23%
Coronary heart disease	0.80%	Ventricular tachycardia	0.25%
HDL cholesterol	1.13%	Diabetes mellitus, noninsulin-dependent	0.32%
Type 2 diabetes	1.48%	Coronary artery disease, susceptibility to	0.51%
LDL cholesterol	1.82%	LDL cholesterol level qt	0.66%
Total cholesterol	1.94%	Hypercholesterolemia, familial	0.68%
Triglycerides	3.30%	Microvascular complications of diabetes	0.69%
Lipid metabolism phenotypes	3.53%	Atherosclerosis, susceptibility to	0.78%
Metabolic syndrome	3.64%	Obesity, susceptibility to	1.88%
Cardiovascular disease risk factors	4.55%	Diabetes mellitus, type 2, susceptibility to	3.14%

We retrieved a total of 4,732 diseases from EIN_OMIM using TMAO as input. Similar to results based on EIN_OMIM, CVDs and their major risk factors, including *myocardial infarction *(top 0.23%), *ventricular tachycardia *(0.25%), *diabetes mellitus, noninsulin-dependent *(top 0.32%), and *coronary artery disease, susceptibility to *(top 0.51%), were ranked highly. Even though the diseases from EIN_GWAS (mainly common complex diseases) and from EIN_OMIM (mainly rare Mendelian disorders) are largely complementary, the high rankings of CVDs and their major risk factors among TMAO-related diseases retrieved from both networks confirmed recent studies and validated our network-based approach in finding TMAO-related diseases.

### Colorectal cancer is highly related to TMAO

Table 4 shows top ten TMAO-related diseases/traits retrieved from EIN_GWAS and from EIN_OMIM. Colorectal cancer was ranked at top 1 (top 0.02%) among 4732 diseases retrieved from EIN_OMIM and at top 10.6% among the 882 retrieved diseases/traits. The high rankings of colorectal cancers based on both networks provided strong evidence supporting our hypothesis that TMAO may be involved in colorectal cancer pathogenesis. Since the GWAS catalog mainly contains common complex diseases/traits and the OMIM database mainly contains Mendelian diseases, the top-ranked diseases retrieved from the two disease networks are quite different. The top ten TMAO-related diseases/traits from EIN_GWAS included several CVD-related risk factors, including *obesity-related traits, metabolite levels, coronary heart disease, metabolic traits*, and *HDL cholesterol*. Interestingly, three autoimmune diseases including *inflammatory bowel disease, multiple sclerosis*, and *Crohn's disease *were also ranked within top ten. The relationship between TMAO and autoimmune diseases warrants further investigation.

**Table 4:** Top ten TMAO-related diseases/traits retrieved from EIN_OMIM and from EIN_GWAS.

Rank	Diseases/traits from EIN_GWAS	Diseases from EIN_OMIM
1	Obesity-related traits	**Colorectal cancer, somatic**
2	Height	Breast cancer, somatic
3	Igg glycosylation	Gastric cancer, somatic
4	Metabolite levels	Ovarian cancer, somatic
5	Inflammatory bowel disease	Schizophrenia, susceptibility to
6	Multiple sclerosis	Asthma, susceptibility to
7	Coronary heart disease	Leukemia, acute myeloid
8	Crohn's disease	Bladder cancer, somatic
9	Metabolic traits	Malaria, cerebral, susceptibility to
10	HDL cholesterol	Thyroid carcinoma, follicular, somatic

Strikingly, among top ten TMAO-related diseases retrieved from EIN_OMIM, seven are cancers, including CRC, breast cancer, gastric cancer and leukemia. Because of the strong (causal) disease-gene associations in the large OMIM database, the observed strong relationship between TMAO and cancers implies that TMAO might be genetically involved in not only CRC but also cancers in general, which we further confirmed in the next section.

### Cancers and metabolic syndromes are highly related to TMAO in general

**Figure 3:**
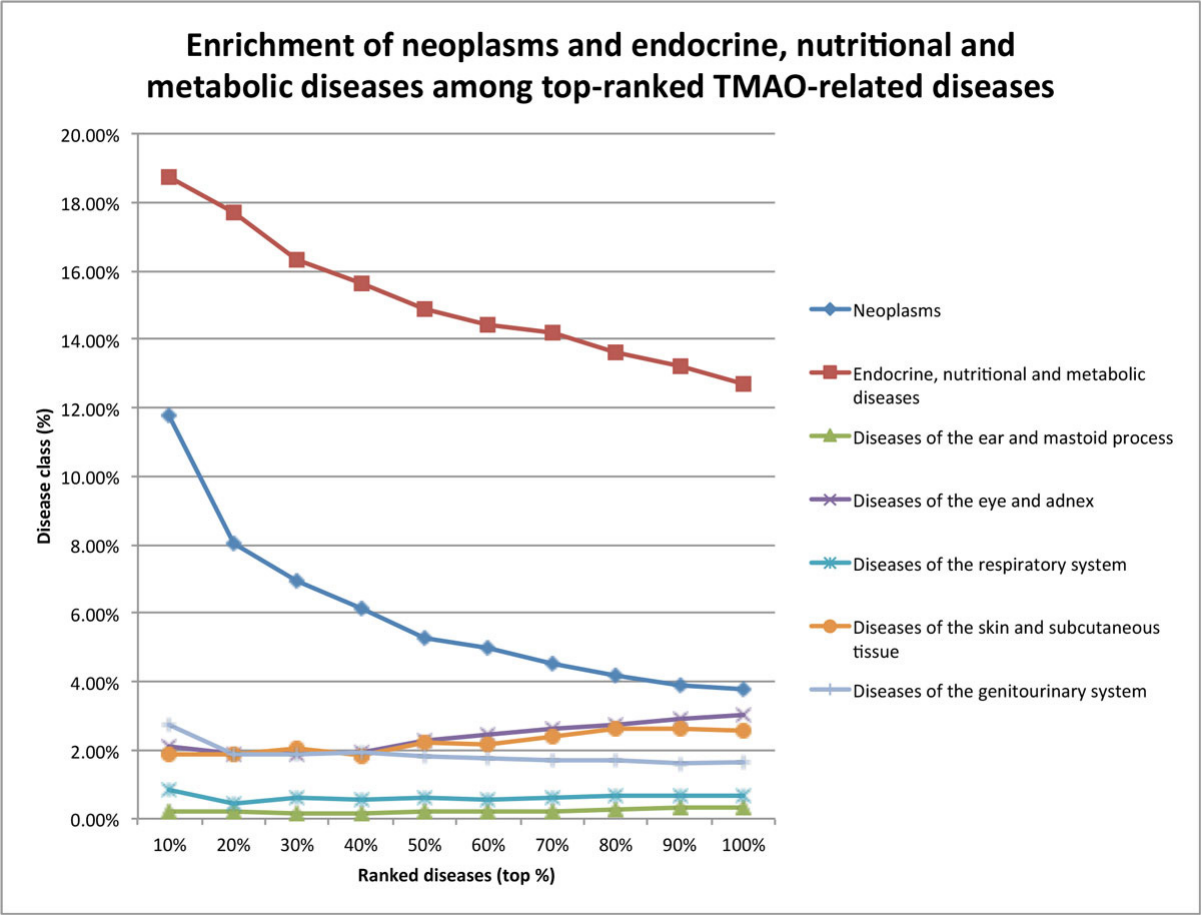
**Disease enrichment analysis for TMAO-related diseases at 10 different ranking cutoffs**. Enrichment of disease classes among TMAO-related diseases at ten ranking cutoffs. TMAO-related diseases were retrieved from the OMIM-based network. The other nine disease classes (not shown) were not enriched among top-ranked diseases.

We examined the distributions of sixteen disease classes among 4,732 TMAO-related diseases retrieved from EIN_OMIM at 10 different ranking cutoffs (top 10%, 20%,

. . . 100%). Among the sixteen disease classes, only two disease classes were enriched among top-ranked TMAO-related diseases: *Neoplasms *and *Endocrine, nutritional and metabolic diseases *(Figure 3). For example, 11.79% of the top 10% ranked diseases were neoplasms, representing a significant 211.9% increase as compared to 3.79% of neoplasms among all retrieved diseases. Similarly, a total of 18.74% of the top 10% ranked diseases were metabolic diseases, representing a 47.8% increase as compared to 12.68% among all retrieved diseases. Given the limited number of diseases contained in the GWAS catalog, we did not perform disease enrichment analysis on diseases/traits retrieved from EIN_GWAS.

### Putative genetic pathways linking CRC to TMAO

We demonstrated that CRC was highly related to TMAO in afore-mentioned sections. We next investigated common genetic pathways that are involved in both TMAO and CRC. The 54 TMAO-associated human genes are involved in a total of 170 pathways. The 53 CRC genes based on OMIM genetics are involved in 503 pathways and the 65 CRC genes based on GWAS studies are associated with 182 pathways. Although no specific genes are shared between TMAO and CRC, many common genetic pathways are associated with both: 52 common pathways between TMAO and CRC based on OMIM genes and 39 common pathways based on GWAS genetics (Table 5).

**Table 5:** Numbers of shared genes and pathways between TMAO and CRC.

	Genes (n)	Pathways (n)
TMAO	54	170
CRC (OMIM)	53	503
CRC (GWAS)	65	182
CRC (OMIM) ∩ CRC (GWAS)	0	118
TMAO ∩ CRC (OMIM)	0	52
TMAO ∩ CRC (GWAS)	0	39
TMAO ∩ CRC (OMIM) ∩ CRC (GWAS)	0	20

Even though there is no overlap between the 53 CRC-associated genes identified from OMIM and the 65 CRC-associated genes identified from the GWAS catalog, these genes shared 118 pathways, which we used to identify genetic pathways linking CRC and TMAO. We found that TMAO shared 20 pathways of these 118 CRCrelated pathways with CRC. The top 10 ranked common pathways between TMAO and CRC (OMIM), TMAO and CRC(GWAS), and TMAO and CRC-genes from both OMIM and GWAS are shown in Table 6.

**Table 6:** Top ten ranked genetic pathways shared between TMAO and CRC.

**TMAO **∩ **CRC (OMIM)**	**TMAO **∩ **CRC (GWAS)**	**TMAO **∩ **CRC (OMIM) **∩ **CRC** (GWAS)
Pathways in cancer Immune system Neurotrophin signaling pathway Cell cycle Wnt signaling CMYB pathway Adaptive immune system MYC activpathway MARK signaling pathway Hemostasis	Immune system Cell cycle Pathways in cancer Metabolism of proteins TCA cycle and respiratory electron transport MYC activpathway Adaptive immune system WNT signaling MAPK signaling pathway Metabolism of lipids and lipoproteins	Immune system Cell cycle Pathways in cancer WNT signaling Adaptive immune system MYC activpathway MAPK signaling pathway Chromosome maintenance Telomere maintenance Metabolism of lipids and lipoproteins

## Discussion

Recent studies have shown a mechanistic link between TMAO, gut microbial metabolism of dietary meat and fat, and risk of cardiovascular diseases (CVDs), and established an obligatory role of gut microbiota in the generation of the proatherosclerotic TMAO from dietary L-carnitine and phosphatidylcholine, abundant in red meat and dietary fat respectively [11-14]. Employing a genome-wide systems analysis approach, we confirmed the association of TMAO with CVDs and other related metabolic disorders such as dyslipidemia. Indeed, inhibition of reverse cholesterol transport has been identified as an important mechanism by which TMAO promotes atherosclerosis [11,13]. Although in vitro and in vivo study data linking TMAO to CRC is still lacking, our present study revealed a striking strong association between TMAO and CRC, and TMAO appears to be involved in many genetic pathways clearly implicated in cancer in general and colon carcinogenesis in particular.

High red meat and animal fat intakes have been well established as risk factors for both CVDs and colorectal cancer. The discovery of the TMAO-CVDs connection mediated by gut microbial metabolism provides evidence for a novel mechanism by which human gut microbiota may influence health and disease. Gut microbiota has long been postulated to modulate risk of CRC. Although increasing evidence shows gut microbial community differences in patients with and without colorectal neoplasia [8-10], the exact mechanisms by which gut microbiota may affect colon carcinogenesis is unknown. Our current study, motivated by the similarity of CVDs and CRC in risk association with dietary red meat and fat consumption suggests that TMAO may also be an important and unappreciated intermediate linking red meat and fat intakes and gut microbiota metabolism to the development of CRC. In vitro and in vivo data directly linking TMAO, gut microbial metabolism of meat and fat to CRC is still lacking. Results from our present study thus shall be only considered as hypothesis generating and warrant further investigations.

## Conclusions

In this study, we present an unbiased data-driven network-based approach to uncover genetic links between TMAO and CRC by integrating and reasoning over vast amounts of disease genetics, protein interactions, and interactions of chemicals and proteins. Our approach is generic and can be readily retargeted to discover novel genetic links among any diseases and chemicals. Our genome-wide analysis demonstrates that systems approaches hold promise for the discovery of novel disease genetic basis. Our results show that TMAO is genetically associated with CRC. This study suggests that TMAO may be an important intermediate marker linking dietary meat and fat and gut microbiota metabolism to risk of CRC, underscoring opportunities for the development of new gut microbiome-dependent diagnostic tests and therapeutics for CRC.

## Competing interests

The authors declare that they have no competing interests.

## Authors' contributions

LL: initiated the hypothesis. RX and QW: jointly designed and implemented algorithms, and performed the experiments. RX, QW, and LL: wrote the paper.

## Declarations

Publication charges for this article have been funded by the Training grant in Computational Genomic Epidemiology of Cancer (CoGE) (R25 CA094186-06).

This article has been published as part of *BMC Genomics *Volume 16 Supplement 7, 2015: Selected articles from The International Conference on Intelligent Biology and Medicine (ICIBM) 2014: Genomics. The full contents of the supplement are available online at http://www.biomedcentral.com/bmcgenomics/supplements/16/S7.
